# Acute and chronic cardiovascular responses to concentric and eccentric exercise in older adults with knee osteoarthritis

**DOI:** 10.1186/s13102-023-00708-1

**Published:** 2023-08-01

**Authors:** Heather K. Vincent, Sharareh Sharififar, Christian McLaren, James May, Kevin R. Vincent

**Affiliations:** Department of Physical Medicine and Rehabilitation, College of Medicine, University of Florida, UF Orthopaedics and Sports Medicine Institute, PO Box 112730, Gainesville, FL 32611 USA

**Keywords:** Resistance exercise, Knee, Osteoarthritis, Cardiovascular, Blood pressure

## Abstract

**Purpose:**

Muscle contraction type in resistance exercise training may confer benefits besides strength in individuals with osteoarthritis and cardiovascular disease (CVD) risks. The purpose of the study was to explore whether Eccentric-resistance training (RT) improved hemodynamic responses to acute walking exercise stress compared to Concentric-RT among individuals with knee OA over four months.

**Methods:**

This was a secondary analysis from a randomized, controlled, single-blinded study. Participants (*N* = 88; 68.3 ± 6.4 yrs; 67.4% female) were randomized to one of two work-matched resistance training (RT) programs against a non-RT control group. Pre-training and month four, participants completed a self-paced Six-Minute Walk Test (6MWT) and progressive treadmill exercise test. Heart rates, blood pressures and mean arterial pressures (MAP) were captured during each test. Antihypertensive medications use was documented at each time point.

**Results:**

Leg strength improved in both training groups by month four (*p* < .05). Changes in 6MWT distance and progressive treadmill test time were not different across groups over four months. Neither Concentric or Eccentric RT produced different hemodyamic responses during the 6MWT compared to the control group post-training. However, Concentric RT was associated with 6.0%-7.4% reductions in systolic blood pressure during the graded treadmill walking test at 50%, 75% and 100% of the test time compared to Eccentric RT and the controls (*p* = .045). MAP values were lower at 75% and 100% of the treadmill test after Concentric RT (5.7%-6.0% reductions) compared to Eccentric RT (1.0%-2.4% reductions) and controls (1.5% and 4.0% elevations) post-training (*p* = .024). Antihypertensive medication use did not change in any group.

**Conclusions:**

The repeated, progressive exposures of Concentric RT-induced blunted the hypertensive responses to acute exercise compared to Eccentric-RT. Among people with knee OA, Concentric-RT may confer strength benefits to manage OA and possibly reduce cardiovascular stress during exercise.

## Introduction

Knee osteoarthritis (OA) affects more than 641 million individuals globally [1], and the prevalence is only expected to increase over the next decade [2]. OA produces pain, elevates inflammation, impairs physical function and contributes to disability. OA also increases the risk of comorbid diseases such as cardiovascular disease (CVD) and its sequelae [3]. CVD is the most common comorbid condition in individuals with knee OA [4], and OA radiographic severity is related to elevated heart rates [5]. Hypertension is also present in 63% of individuals with knee OA [6]. Progression of OA, and worsening of radiographic and symptomatic features are related to presence of CVD [7, 8]. Knee symptoms interfere with ambulation and participation in physical activity [9], and there are associations between sedentariness, OA symptoms [10] and CVD risks [11]. Safe, tolerable and integrated strategies that a). simultaneously manage OA progression and CVD risks, and b). attenuate acute hemodynamic responses during ambulatory activities like brisk walking, are needed to positively shift the health trajectory and prolong functional independence in this population.

Resistance training (RT) is a training modality that contributes to skeletal muscle adaptation and gains in strength, and is a key clinical component of knee OA management [12]. During exercise, muscles contract concentrically, eccentrically, or a combination of these two muscle actions depending on the movement type and equipment used. Both concentric and eccentric resistance exercise improve leg strength, reduce knee pain and discomfort with functional tasks and walking in older adults with knee OA, and both muscle contraction modes are well-tolerated and safe [13, 14]. With respect to the cardiovascular system, acute concentric resistance exercise produces greater stress (heart rates, blood pressures, ventilation) than eccentric exercise [15–19]. Consequently, some investigators have proposed that the uncoupling of skeletal muscle load and cardiovascular stress that occurs with eccentric strengthening exercise might allow older adults to ‘safely’ participate in intensive strength training, especially if risks for adverse cardiopulmonary events are present [15, 20]. However, concentric resistance exercise training that chronically exposes the older adult to elevated hemodyamic responses may actually translate to more favorable chronic cardiovascular adaptations than eccentric training but this has not yet been tested. In our earlier work, we found that concentric resistance exercise training blunted elevations in mean arterial pressure, diastolic blood pressure and heart rates during progressive walking exercise bouts, with faster hemodynamic recovery compared to untrained older adults. Importantly, better hemodynamic results were found with high intensity training compared to low intensity training [21]. It remains unclear which contraction type best improves cardiovascular adaptation and hemodynamic responses to walking activity. The intensities of walking exercise can vary from light to vigorous and are commensurate with stresses of daily life ambulatory activities, such as stair climbing, walking on a street uphill, or rushing to catch a train [22]. Improved blood pressure control during physical activity has prognostic implications for weathering ambulatory stressors in daily life, for reducing CVD morbidity and for lowering mortality risk [23].

Hence, the purpose of the study was to determine whether Eccentric-RT improved hemodynamic responses to acute walking exercise stress compared to Concentric-RT among individuals with knee OA over four months. Given that evidence in acute exercise studies demonstrated that Eccentric-RT invoked less cardiovascular stress, we hypothesized that Eccentric-RT would produce more favorable hemodynamic responses to acute walking exercise bouts than Concentric-RT after the intervention period.

## Methods

### Design

This research is a secondary analysis of a four-month randomized, controlled, single-blinded study of two differing, work-matched resistance training (RT) protocols (concentric and eccentric controlled) against a non-RT control group. This study followed the Consolidated Standards of Reporting Trials (CONSORT) 2010 guidelines for reporting parallel group randomized trials [24]. The study was registered as a clinical trial NCT00187863.

## Participants and screening

### Recruitment

Individuals with knee osteoarthritis were recruited using flyers and newspaper listings distributed in north-central Florida, surrounding the Gainesville area, using the UF Orthopedic & Rehabilitation clinics, research mailing list provided by the UF Claude Pepper Aging Center, and Clinical Trial Register. All study measures were collected at the University of Florida Human Dynamics Laboratories.

### Eligibility criteria

**Fig. 1:**
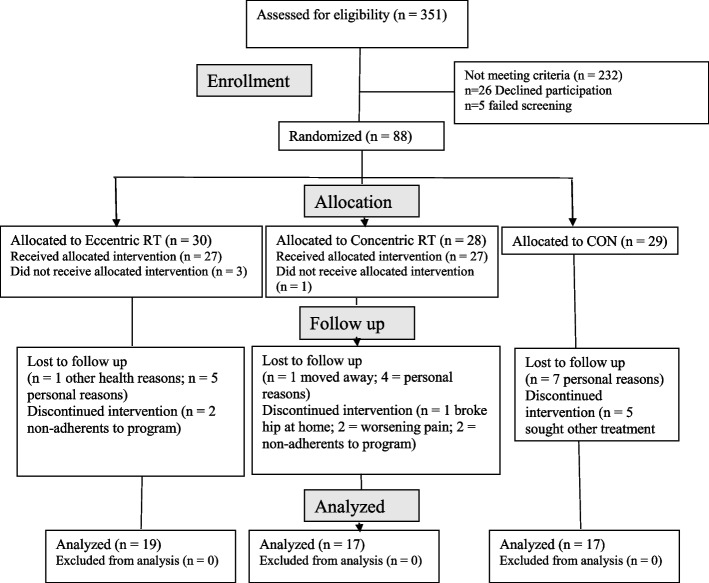
CONSORT diagram of the study flow

The screening process included initial review of individual’s eligibility criteria by the study coordinator and study physician. This study was approved by the University of Florida Institutional Review Board, and all procedures on human subjects were conducted in accordance with the Helsinki Declaration of 1975, as revised in 1983. All participants provided written, informed consent to participate. The CONSORT study flow diagram is shown in Fig. 1.

### Inclusion criteria

Individuals fit inclusion criteria if they were aged 60–85 years, had weightbearing anterior–posterior radiographic imaging demonstrating Kellgren and Lawrence OA grade two or three for the affected knee [25], presence of ongoing (= 6 months) osteoarthritis of the knee using American College of Rheumatology criteria [26], knee pain from tibiofemoral OA rather than patellofemoral OA, able to actively participate in regular exercise for four months, have no other musculoskeletal conditions limiting resistance exercise participation, and free from abnormal cardiovascular responses to exercise.

### Exclusion criteria

Individuals were excluded from the study if they had any unilateral knee surgery within the last 12 months, symptoms consistent with lumbar radiculopathy or vascular claudication, anterior knee pain due to diagnosed isolated patella-femoral syndrome or chondromalacia in either knee primarily, have had a corticosteroid or hyaluronic acid injections administered within three months of study participation, have added new over-the-counter or prescription pain medication within two months of study participation.

### Screening

If a participant was deemed eligible to enroll in the study they were invited to the testing facility to be further screened using a walking graded treadmill test. If the participant was free from abnormal cardiovascular response during and following treadmill testing they were eligible to continue in the research study. A symptom-limited, modified Naughton treadmill test protocol was administered at baseline and post-intervention to all study groups. All testing sessions abided by the American College of Sports Medicine guidelines with electrocardiogram heart monitoring and blood pressure measures [27]. Open-circuit spirometry was used to determine the rate of oxygen use and carbon dioxide production using a metabolic cart (VIASYS; CareFusion Corp, San Diego, CA).

#### Randomization and blinding

Participants were each assigned to one of the following study groups: a concentric-controlled exercise (Concentric RT), an eccentric-controlled (Eccentric RT), and a non-RT waitlist control group (CON). Patient were randomized using a computer-generated list with hidden assignment of individual participants. There was one designated study coordinator to distribute individual participant study group assignment. These assignments were distributed to the participants in sealed, numbered envelopes. The principal investigator and testers who collected study data were blinded from group allocation. All training sessions were conducted by experienced study coordinators and exercise physiologists.

#### Resistance exercise training

Both the Concentric RT and Eccentric RT groups completed scheduled training session using MedX® clinical resistance exercise machines. All study participants received a health education packet, which provided information regarding healthy behaviors (Centers for Disease Control Physical Activity for Everyone and Nutrition for Everyone; American Heart Association Physical Activity in Daily Life).

Both RT intervention groups performed two training sessions per week (total of 32 sessions). Each training session consisted of a five-minute warm up on a treadmill or stationary cycle. A single set of 12 repetitions for the following exercises at an intensity of 60% of 1-RM was performed in the following order: leg press, knee extension, knee flexion, chest press, seated row, shoulder press, biceps curl and calf press. Perceived level of exertion was rated using the 6–20 point Borg Scale [28]. The repetition structure on the eccentric exercise machines and comparative concentric exercise machines were modified to equalize the work performed on a given exercise between the study groups [14]. 1-RM was adjusted based upon each participant’s level of perceived exertion each week per exercise to maintain the RPE value between 17–18 for the duration of the study. Rest intervals between sets were three minutes in duration. Strength gains were expressed as a percentage change from pre-post training.

Participants assigned to the CON group were advised to resume normal activities and follow-up in four months, after the study intervention had concluded. CON participants were offered to complete resistance training sessions (Concentric RT and Eccentric RT) following conclusion of the research study. Furthermore, CON group participants were contacted weekly by telephone to promote adherence to the health education pamphlet given to all participants. All baseline strength and functional tests were supervised and reviewed by the study physician.

## Cardiovascular responses to walking exercise

Two walking tests were performed to represent sustained self-paced walking and progressively intensive walking.

### Self-paced six-minute walk test (6MWT)

The 6MWT was performed with no assistive devices along a 30-m hallway. All testing procedures were conducted in accordance to Osteoarthritis Research Society International Standards [21]. Distance markers were placed along the hallway every meter. Each participant was given the same instructions to cover as much ground as fast as they could. Encouragement was provided by a study coordinator at each end of the hallway. Heart rates (HR) were continuously monitored and reported at rest, one-minute intervals during the test and to three minutes posttest using a HR monitor (Polar Electro, USA). For the 6MWT, blood pressures were collected before the walk at rest and after the walk test during recovery at minutes 1 and 3. Resting and post-exercise measures were performed in seated position. Change scores in MAP with the intervention were determined from the difference in baseline value to month four value. Before the test at rest and at one-minute intervals, knee pain symptoms were collected at the end of each minute of the test using the 11-point Numerical Pain Rating scale (NRS_pain_; where 0 = no pain and 10 = worst imaginable pain). The NRS_pain_ has good psychometric properties is valid in knee OA and has moderate-to-large responsiveness with treatments [29].

### Progressive treadmill walking test

The initial screening Naughton test described earlier was conducted before the four-month intervention. If the participant screened eligible with no cardiovascular issues, this Naughton test was used as the pre-training measure. This same protocol was repeated after the four-month intervention. the modified Naughton treadmill test protocol Open-circuit spirometry was used to determine the rate of oxygen use and carbon dioxide production using a metabolic cart (VIASYS©; CareFusion Corp., San Diego, CA). Blood pressures and heart rates were collected at rest, the final minute of each test stage, at minute six, and posttest minutes one and three. Exercise blood pressures have emerged as a method to identify individuals with high pressure that might have previously gone undetected in clinic [22]. Heart rates were obtained from the electrocardiogram system (Quinton Stress Testing System; Welch Allyn, Skaneateles Falls, NY). For both tests, systolic and diastolic blood pressures were used to calculate mean arterial pressures (MAP) as follows: MAP (mmHg) = (Systolic blood pressure + 2[Diastolic blood pressure])/3. To standardize when responses were presented here, blood pressure, HR and MAP were presented at baseline, 25%, 50%, 75% and 100% of the individual participant treadmill test time [21]. Resting and post-exercise blood pressure measures were performed in a static standing position, and during recovery walking, respectively. Metabolic equivalent levels (METs) at the quartiles of test time were obtained from the metabolic cart output, where 1MET = 3.5 ml/kg*min.

### Blood pressure medication use

Participants recorded the number and type of any medications used to manage hypertension at baseline and month four in a study log.

### Statistics

Statistics were conducted in IBM SPSS Version 28.0 (Armonk, NY). Unless otherwise specified, data are expressed as means ± standard deviation (SD). Differences in baseline categorical measures across concentric (Concentric RT), eccentric (Eccentric RT), and control (CON) groups were assessed using ?^2^ tests. Differences in baseline continuous measures across study groups were assessed with analysis of variance, using the Tukey–Kramer test for pairwise comparisons, which also adjusted for multiple comparisons using the Bonferroni method. Non-normally distributed measures were log transformed prior to analyses. Differences between groups for baseline continuous variables were examined using a one-way analysis of variance with a Tukey post hoc test. Per-protocol analysis (inclusion of participants who did not have any violations to the study protocol) were performed here. Data were analyzed using general linear models. These models included three factors: time in the walking test (time in test [minute]); time point in study [pretraining at Baseline, post-training at Month 4]) and study group (CON, Concentric RT, Eccentric RT) as main effects, with a three-way interaction model between time in test, time in study and group. Covariates in the models included age and presence of knee pain in one or both knees. A significant time (test) × time in study × group interaction would indicate that the change in outcome from pretraining to post-training differed among groups during each walking test. Moreover, we performed multivariate tests with multiple comparisons analysis and used Tukey’s HSD post hoc tests to determine where the differences in cardiovascular responses occurred during the walking tests. Dependent variables were HR, blood pressures and MAP, and independent variable was study group (CON, Concentric RT, Eccentric RT). Cohen’s *d* effect sizes were calculated, where = 0.2 was a small effect, = 0.5 was a medium effect and = 0.8 represents a large effect size.

### Sample size

Sample size was previously determined based on knee pain subscore improvements on the Western Ontario McMaster University Index [14]. A 30% reduction in the WOMAC pain subscore was used to estimate the sample size of 20 completers per group with a power of 0.80 and a level of 0.05.

## Results

### Participants

Table 1 provides the characteristics of the three study groups at baseline. Overall, participants were well-matched for sex, race, knee pain symptoms and self-reported activity levels. The Eccentric RT group had fewer participants with hypertension than the other groups (*p* < 0.05). Of the original 90 participants, dropouts were as follows: In the CON group, 7 withdrew for personal reasons such as lost interest in waiting to participate, 5 sought other knee pain treatments and didn’t want to wait for the intervention period to end. In the Concentric RT group, 1 broke hip at home and withdrew; 2 developed worsening knee pain; 4 withdrew for personal reasons such as lost interest or not enough time; 1 moved away from area; 2 were withdrawn by study team due to lack of protocol training adherence. In the Eccentric RT group, 1 developed cancer and withdrew; 5 had other personal reasons such as loss of interest or could not commit time; 2 were withdrawn by the study team for failing to remain adherent to the training program.

**Table 1:** Participant characteristics at baseline. Values are means ± SD (95% CI)

	Control	Concentric RT	Eccentric RT	*p*
	(*n* = 28)	(*n* = 27)	(*n* = 30)	
Age	68.6 ± 7.1 (66.0–71.2)	69.5 ± 6.5 (66.9–72.1)	66.8 ± 5.4 (64.8–68.9)	.287
Sex, female (#, %)	21 (63.6)	18 (66.6)	21 (7.0)	.931
Race (#, %)				
African-American	2 (6.0)	3 (11.1)	2 (6.7)	2 (6.0)
Hispanic	2 (6.0)	0 (.0)	0 (.0)	
Caucasian	27 (82.0)	23 (85.2)	28 (93.3)	
Other	2 (6.0)	1 (3.7)	0 (.0)	.326
BMI (kg/m^2^)	3.1 ± 6.2 (27.8–32.4)	32.8 ± 7.4 (29.9–35.7)	28.7 ± 6.6 (26.2–31.1)	.069
Duration of knee pain (yr)	7.9 ± 8.9 (4.7–11.3)	7.8 ± 8.2 (4.5–11.1)	12.8 ± 12.0 (8.3–17.2)	.100
Pain in knees (#, %)				
One	13 (39.4)	9 (33.3)	11 (36.7)	
Both	20 (6.6)	18 (66.7)	19 (63.3)	.942
Walking knee pain (#, % yes)	18 (54.5)	16 (59.2)	18 (6.0)	.894
Walking = 3 times per week (#, %)	11 (33.3)	7 (25.9)	10 (33.3)	.738
Comorbid conditions (#, %)				
Obesity	11 (33.3)	6 (22.2)	11 (36.7)	.476
Heart disease	2 (6.0)	1 (3.7)	1 (3.3)	.852
Hypertension	19 (57.6)	14 (51.9)	8 (26.7)	.037
Using antihypertensive medication (#, %)				
	8 (24.2)	9 (33.3)	8 (26.7)	.673

*RT* resistance training, *BMI* body mass index

### Strength gains

**Fig. 2:**
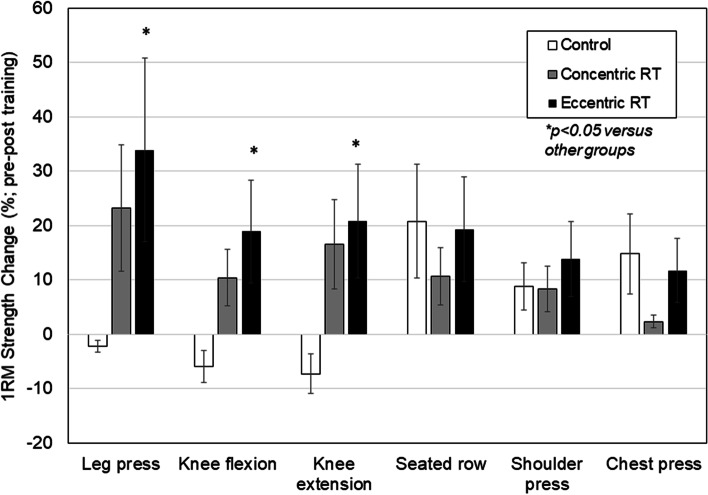
Relative changes in 1-repetition maximum (1RM) in individuals with knee OA from pre-post training. Values are expressed as percent change and are reported as means ± SD

Relative 1-RM strength changes ranging from 1.4%-33.9% occurred in both training groups from pre-post training for the leg press, knee flexion and knee extension compared to 2.2%-7.3% strength losses in the CON (Fig. 2; all *p* < 0.05). Percentage strength gains in 1-RM for these leg exercises were 4.3%-1.7% higher in the Eccentric RT than the Concentric RT (all *p* < 0.05; Effect size range *d* = 0.23 – 0.71). There were no significant differences in gains in strength for the chest press, seated row or shoulder press among the three study groups over four months (all *p* > 0.05).

### Blood pressure medication use

At baseline, the CON**,** Concentric RT and Eccentric RT groups reported using 0.8 ± 1.1, 0.9 ± 0.9 and 0.8 ± 1.0 blood pressure medicines, respectively. At month four, there was no significant reduction in the number of medications used or the proportion of participants using these medications by study group (all *p* > 0.05). Seven participants reported using antihypertensive medicines in each group at month four.

### 6MWT responses

**Fig. 3:**
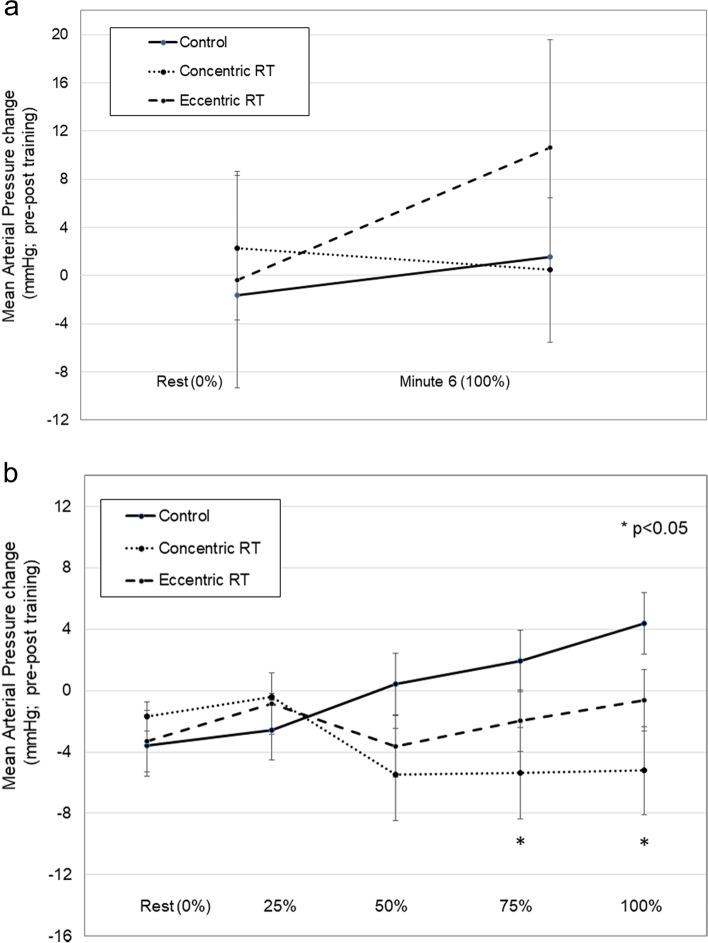
**a** and **b** Changes in mean arterial pressure (MAP) from pre-post training during a progressive treadmill test (**a**) and during a Six-Minute Walk Test. with Eccentric RT, Concentric RT or with a no training control condition (CON) (**b**). Values are expressed as percent change and are reported as means ± SD. * denotes significant difference versus CON and Eccentric RT at *p* < .05

Walking distances at pre-training and month four were as follows: 472 ± 86 m and 467 ± 92 m (Concentric RT), 536 ± 95 m and 558 ± 102 m (Eccentric RT), and 537 ± 118 m and 560 ± 124 m (Control group; *p* = 0.375). Blood pressures, HR and walking pace for the 6MWT are shown in Table 2. There were no statistically significant group by time interactions for systolic blood pressure, diastolic blood pressure, HR, peak knee NRS_pain_ or walking pace. The pre-post training MAP changes are shown in Fig. 3a, and these were not different among groups.

**Table 2:** Blood pressure, heart rate responses and pace during a 6-min walk test (6MWT). Values are means ± SD. *intxn* = interaction of time in walking test (% of test), time in study (Baseline to Month 4) and study group (control, Concentric and Eccentric RT)

	Control		Concentric RT		Eccentric RT		*p*
	Baseline	Month 4	Baseline	Month 4	Baseline	Month 4	intxn
SBP (mmHg)							
Rest	127 ± 18	125 ± 19	126 ± 19	130 ± 13	124 ± 15	127 ± 11	
Minute 6	140 ± 121	137 ± 18	146 ± 17	140 ± 18	150 ± 19	148 ± 13	
1-min post	140 ± 20	135 ± 17	140 ± 24	137 ± 18	137 ± 11	145 ± 18	
3-min post	127 ± 18	127 ± 22	134 ± 25	128 ± 12	128 ± 10	134 ± 17	.604
DBP (mmHg)							
Rest	75 ± 9	72 ± 8	75 ± 12	80 ± 8	76 ± 9	75 ± 8	
Minute 6	77 ± 10	75 ± 7	73 ± 12	74 ± 12	81 ± 14	81 ± 9	
1-min post	79 ± 10	76 ± 8	75 ± 12	76 ± 12	80 ± 12	80 ± 11	
3-min post	75 ± 8	72 ± 10	74 ± 12	78 ± 6	78 ± 10	80 ± 7	.530
HR (bpm)							
Rest	72 ± 9	74 ± 10	76 ± 11	80 ± 12	75 ± 10	77 ± 11	
Minute 1	103 ± 19	106 ± 21	101 ± 17	105 ± 10	107 ± 11	109 ± 17	
Minute 2	111 ± 19	113 ± 19	111 ± 25	111 ± 10	113 ± 14	121 ± 16	
Minute 3	113 ± 19	116 ± 18	114 ± 24	120 ± 30	116 ± 14	123 ± 16	
Minute 4	115 ± 20	117 ± 18	115 ± 25	122 ± 30	117 ± 15	125 ± 18	
Minute 5	115 ± 20	118 ± 19	115 ± 24	114 ± 11	118 ± 16	126 ± 17	
Minute 6	116 ± 19	122 ± 22	116 ± 24	114 ± 10	118 ± 17	128 ± 18	
1-min post	92 ± 18	94 ± 18	92 ± 17	95 ± 11	93 ± 17	101 ± 14	
3-min post	80 ± 11	83 ± 12	81 ± 15	85 ± 12	83 ± 14	87 ± 13	.828
Peak NRS_pain_	2.2 ± 1.6	1.8 ± 1.2	3.0 ± 2.5	1.5 ± 2.1	2.1 ± 1.7	1.3 ± 1.6	.404
Walking Pace (m/s)							
Minute 1	1.3 ± .4	1.5 ± .3	1.2 ± .2	1.2 ± .2	1.4 ± .3	1.5 ± .2	
Minute 6	1.2 ± .5	1.4 ± .3	1.1 ± .3	1.1 ± .2	1.3 ± .3	1.4 ± .3	.986

### Progressive treadmill test responses

Exercise test times at baseline and month four were as follows: 1.0 ± 2.2 min and 11.2 ± 3.7 min (Concentric RT), 14.4 ± 5.9 min and 15.5 ± 4.7 min (Eccentric RT) and 12.5 ± 4.2 min and 13.3 ± 5.1 min (Control group; *p* = 0.935). Peak MET levels achieved during the test did not change in the CON from baseline to month four (8.0 ± 1.6 to 7.9 ± 2.4), but increased in the Concentric RT (6.6 ± 0.9 to 7.0 ± 1.7) and in the Eccentric RT group (8.4 ± 2.5 to 9.4 ± 2.2). Blood pressure and HR responses to this progressive treadmill exercise are shown in Table 3. A significant 3-way interaction existed for systolic blood pressure. Tukey post-hoc tests revealed that the Concentric RT group demonstrated reductions in systolic pressures during the treadmill test compared to Eccentric RT at month four at 100% of the test (*p* = 0.045; Effect size 0.78). There were no statistically significant group by time interactions for diastolic blood pressure or HR responses, peak knee NRS_pain_ or fastest walking pace. The changes in MAP from baseline to month four are shown in Fig. 3b. The Concentric RT group demonstrated greater four-month reductions in MAP than both the CON and Eccentric RT groups during the treadmill test (*p* < 0.05; Effect sizes were 1.04 and 0.48, respectively); Tukey HSD post hoc test *p* values for 75% and 100% of the treadmill test were significant (both *p* < 0.05; Effect size range *d* = 0.50 – 1.3).

**Table 3:** Blood pressure and heart rate responses to progressive maximal treadmill exercise at baseline and after four months of resistance exercise training. Values are means ± SD. *intxn* = interaction of time in walking test (% of test), time in study

	Control		Concentric RT		Eccentric RT		*p*	post hoc
	Baseline	Month 4	Baseline	Month 4	Baseline	Month 4	intxn	specific group differences
SBP (mmHg)								
Rest	122 ± 13	120 ± 11	125 ± 17	126 ± 15	127 ± 12	122 ± 17		
25% of test	138 ± 16	136 ± 12	141 ± 16	146 ± 36	145 ± 12	137 ± 18		
50% of test	150 ± 17	152 ± 16	162 ± 24	150 ± 20	160 ± 14	153 ± 17		
75% of test	164 ± 22	168 ± 20	175 ± 27	162 ± 22	169 ± 15	168 ± 16		
100% of test *	173 ± 26	180 ± 21	186 ± 28	177 ± 22	179 ± 19	179 ± 20		*Concentric v Eccentric
One-minute post	157 ± 20	164 ± 24	164 ± 28	164 ± 25	163 ± 16	160 ± 27	.045	
DBP (mmHg)								
Rest	70 ± 11	74 ± 8	74 ± 10	76 ± 7	76 ± 10	74 ± 10		
25% of test	72 ± 11	73 ± 9	75 ± 11	74 ± 8	73 ± 18	74 ± 11		
50% of test	72 ± 11	75 ± 9	76 ± 12	75 ± 8	75 ± 12	75 ± 10		
75% of test	73 ± 11	77 ± 10	76 ± 13	75 ± 10	76 ± 13	75 ± 11		
100% of test	72 ± 12	78 ± 10	78 ± 13	76 ± 11	76 ± 14	76 ± 12		
One-minute post	71 ± 10	75 ± 10	75 ± 12	75 ± 10	74 ± 11	74 ± 12	.105	
HR (bpm)								
Rest	80 ± 12	85 ± 11	83 ± 12	84 ± 10	82 ± 14	85 ± 13		
25% of test	101 ± 13	101 ± 12	105 ± 13	101 ± 12	102 ± 12	100 ± 13		
50% of test	111 ± 23	116 ± 13	117 ± 15	110 ± 13	118 ± 13	114 ± 14		
75% of test *	128 ± 13	131 ± 14	129 ± 16	121 ± 15	128 ± 26	128 ± 12		
100% of test *	140 ± 15	143 ± 16	139 ± 16	131 ± 15	150 ± 19	143 ± 13		*Concentric v Eccentric
One-minute post	119 ± 15	124 ± 15	121 ± 15	117 ± 16	133 ± 17	128 ± 15	.753	*Concentric v Eccentric, & Control v Eccentric
Peak NRS_pain_	2.2 ± 2.1	1.9 ± 2.1	3.2 ± 1.8	2.1 ± 2.1	2.4 ± 1.3	1.3 ± 1.6	.417	
Walking pace (m/s)	1.3 ± .2	1.2 ± .2	1.2 ± .2	1.5 ± .2	1.2 ± .2	1.3 ± .1	.243	

* denotes the time point and specific group difference at which statistical significance was detected

## Discussion

We explored acute hemodynamic responses to self-paced and progressive walking after four months of either Concentric RT or Eccentric RT and a non-exercise control period. Contrary to our hypothesis, the key findings of our study were that Concentric RT blunted the hemodynamic response to progressive treadmill walking exercise by month four compared to Eccentric RT in people with knee OA, but not during the self-paced 6MWT. While Eccentric-RT may be acutely less stressful to the cardiovascular system, our findings show that the graduated progressive Concentric RT exposures that cause higher cardiovascular stress [15, 16, 18, 19] during the intervention period actually produced protective hemodynamic adaptations over time. Blunting stress-induced hypertension may reduce the likelihood if a cardiac event, but this will require additional longitudinal study.

There are very few comparative studies of work-matched concentric and eccentric strength training on hemodynamic responses in older adults, especially with knee OA. One 16-week study used eccentric resistance exercise combined with aerobic exercise as an intervention in 60 hypertensive women [30]. These authors found 13%-19% reductions in resting systolic and diastolic blood pressures from pre-post training. Other investigations show that concentric RT is metabolically more challenging than eccentric exercise (greater rate of oxygen consumption, blood lactate levels and ventilation rate) [31]. In a work-matched study, concentric RT enhanced post-exercise vasodilation, arterial blood flow and pulse wave velocity and subsequently, lowered blood pressure compared to eccentric RT [32]. While we did not observe significant mean changes in resting blood pressure or HR with either resistance exercise type, Concentric RT produced 4.4% reductions in systolic blood pressure at 3 min post completion of 6MWT compared to the Controls and Eccentric RT (Table 2; Effect sizes *d* = 0.6 and 1.2, respectively). This finding suggests a medium to large effect of improved vascular responsiveness to recovery with acute exercise with Concentric RT. Moreover, Concentric RT reduced systolic blood pressure during the progressive walking exercise by 6.0%-7.4% during moderate to maximal exercise stages of = 5 METS, or 60–80% of resting HR (Table 3). Reductions in MAP averaging 6.5 mmHg also occurred with Concentric RT at 75%-100% of the test, the effect sizes of which were medium to large compared to the other groups (Fig. 3a). The clinical importance of this change is that a 5 mmHg reduction in blood pressure has been shown to be associated with a 10% risk reduction of major cardiac events over time [33]. While we acknowledge that this prior finding was in reference to reductions in resting blood pressure, there is the potential that reductions in blood pressure that occur during vigorous exercise may help lower risk for exercise-induced cardiac issues. For individuals with knee OA who have cardiovascular disease risks, Concentric RT may offer both strengthening-related and cardiovascular protective adaptations. Future research could examine whether the resistance training-induced changes in blood pressure also correspond to stressful hemodynamic adaptations during real-life physical activities such as yard work, stair climbing, walking or performing activities of daily living that reach MET levels of = 5. Long-term studies that vary the Concentric RT exposure, duration and intensity while tracking cardiovascular health would provide evidence of effective dosages necessary to protect against stress-induced adverse cardiac issues in this population.

The fact that there were not statistically significant hemodynamic changes during the self-paced 6MWT suggests that participants likely mitigated cardiovascular responses through controlling speed. Individuals walking overground can easily self-adjust their work load when tired or when OA symptoms become uncomfortable. The 6MWT likely did not produce the physiological stress needed to expose resistance training-induced hemodynamic adaptations. In contrast, individuals walking on a treadmill do so at set speeds and grades and cannot adjust their work loads. Higher cardiovascular stresses were achieved through progressive elevations in treadmill incline and speed. From this finding, researchers may consider using progressive walking tests to reveal hemodynamic responses to resistance training that may not otherwise be detected by self-paced tests.

While we did not specifically measure mechanisms underlying hemodynamic adaptations in this study, a few explanations are offered. First, chronic resistance exercise may improve vascular reactivity, local exercised vessel diameter, and endothelial function, any or all of which may modify total peripheral resistance [34]. There is the possibility that the higher level of metabolic work produced by concentric muscle actions results in greater production of nitric oxide compared to eccentric actions and this warrants further investigation. Second, baroreceptors in the aortic arch and carotid sinuses may adapt and reset following RT stress on the vasculature [34]. Baroreceptors regulate autonomic sympathetic nervous system activity and resultant hemodynamic responses [35]. Given that concentric resistance training induces more profound blood pressure responses than eccentric training, the baroreceptor sensitivity adaptations may be greater which could translate to reductions in systolic and mean blood pressures observed in this study. Third, metabolic and immune responses differ in muscle exercised with concentric and eccentric contractions. Stavres et al. [32] postulated that several mechanisms might be occurring in this domain, including transient GABAergic buffering of baroreceptors and heightened sympathoinhibition with concentric exercise, which reduces HR and ultimately blood pressure. Prospective measures of autonomic function, muscle activation patterns, metabolites and vascular reactivity from pre- to post-training would provide data critical to our understanding of blood pressure adaptations to these contraction types in this population.

### Limitations and strengths

Given the relatively small sample in each group, potential interindividual variability in hemodynamic training adaptations, especially among those with hypertension, may have precluded statistical significance for some outcome measures [36]. This is a secondary exploratory analysis from a study originally powered to detect resistance training differences in knee OA symptoms [14]. As such, this sample size may not have been adequate to detect training group significance for some of the hemodyamic variables. Interestingly and surprisingly, the non-exercise control group improved strength in the upper body muscle groups from pre-training to month four. While this group was provided healthy living printed materials, they were instructed to maintain the same activity patterns until the study was completed. It is possible that in lieu of being randomized to an RT group, these participants engaged in other in healthy activities that fostered strength gains. The strengths of the study include a rigorous study design, intensive individual supervision during the training intervention and objective outcomes. In addition, these findings provide the foundation needed to power subsequent training studies in individuals with knee OA and CVD risks. These data serve as the foundation for future studies determining the Concentric RT prescriptions necessary for optimal hemodynamic adaptation.

## Conclusion

While leg strength gains occurred with both types of resistance exercise, Concentric RT also blunted hemodynamic responses to progressive treadmill walking at intensities = 5 METS compared to Eccentric RT and no exercise. For people with knee OA, this resistance exercise type may offer strength benefits to manage OA and to improve exercise tolerance and possibly reduce cardiovascular stress during exercise.

## Data Availability

Datasets analyzed during the current study are available upon request.
